# Supplementary material for: Exploring 400 Gbps/λ and beyond with AI-accelerated silicon photonic slow-light technology
Source: Nat Commun. 2025 Jul 16;16:6547. doi: 10.1038/s41467-025-61933-5 (PMC12267857; doi:10.1038/s41467-025-61933-5)
Supplement: Supplementary file 1 — Supplementary Information [file 41467_2025_61933_MOESM1_ESM.pdf]

Supplementary Information – Exploring 400 Gbps/λ and beyond with AI-accelerated silicon photonic slow-light technology

Changhao Han[1,2,*], Qipeng Yang[1,*], Jun Qin[3,*], Yan Zhou[4,*], Zhao Zheng[1], Yunhao Zhang[5], Haoren Wang[5], Yu Sun[3], Junde Lu[3], Yimeng Wang[1], Zhangfeng Ge[4], Yichen Wu[1], Lei Wang[5], Zhixue He[5], Shaohua Yu[1,5], Weiwei Hu[1], Chao Peng[1,5,6], Haowen Shu[1,6,§], John E. Bowers[2,‡] and Xingjun Wang[1,4,5,6,†]

[1] *State Key Laboratory of Photonics and Communications, School of Electronics, Peking University, Beijing 100871, China*

[2] *Department of Electrical and Computer Engineering, University of California, Santa Barbara, CA 93106, USA*

[3] *Key Laboratory of Information and Communication Systems, Ministry of Information Industry, Beijing Information Science and Technology University, Beijing 100192, China*

[4] *Peking University Yangtze Delta Institute of Optoelectronics, Nantong 226010, China*

[5] *Peng Cheng Laboratory, Shenzhen 518055, China*

[6] *Frontiers Science Center for Nano-optoelectronics, Peking University, Beijing 100871, China*

[*] *These authors contributed equally to this work*

Corresponding Author:

[§]haowenshu@pku.edu.cn, [‡]bowers@ece.ucsb.edu, [†]xjwang@pku.edu.cn

1 Architecture of the silicon slow-light modulator

The introduction of slow-light waveguides allows the modulator to be miniaturized, so the silicon slow-light modulator (Si-SLM) possesses an ultra-compact footprint. For the overall architecture of the Si-SLM, in order to ensure sufficient modulation depth within the compact modulation arms, the modulator adopts GSGSG-type dual-drive radio-frenquency (RF) electrodes under a Mach-Zehnder (MZ) interferometer structure. The designed dual-drive Si-SLM architecture is shown in Fig. S1a, which demonstrates a cross-section view of the connection relationship between PN junctions and RF electrodes. The device is based on a rib-type silicon optical waveguide structure, fabricated on a 220-nm silicon layer using a standard 90-nm lithography. The PN junction is formed by ion implantation in the silicon waveguide region and three doping concentration levels are adopted here. Low-concentration N/P doping (5.0×10^{17}/cm^3) is utilized to form a PN junction in the waveguide core layer, which can effectively reduce the absorption of the light field by carriers; high-concentration N++/P++ doping (4.0×10^{20}/cm^3) is for ohmic contact between silicon and metal; medium-concentration N+/P+ doping (2.0×10^{18}/cm^3) is used to connect high and low concentration doping regions.

Meanwhile, Fig. S1b demonstrates the top view of the device structure. Symmetrical RF electrodes are used on the two modulation arms. The RF electrode is a coplanar waveguide (CPW) structure, in which the thickness of the Cu electrode is 1.2 μm. In order to achieve characteristic impedance matching, the signal electrode width is set to 10 μm, and the distance between the ground and signal electrodes is 6.4 μm. Meanwhile, on-chip termination resistors are integrated

at the remote end of RF electrodes, with a measured single resistance of around 55 Ω, to achieve better impedance matching and further reduce microwave reflection. In fact, at the electrode design level, there still exists a lot of space for optimization. At present, the microwave refractive index of the electrodes is around 2.1. If the segmented slow-wave electrodes can be adopted, the phase matching with the slow-light waveguide can be improved, thereby further increasing the EO modulation bandwidth of the device[1,2]. Under the dual-drive architecture of GSGSG-type electrodes, the two modulation arms can be operated in push-pull mode by loading differential high-frequency signals with the same time delay but opposite phases, thus enlarging the phase change accumulation. Based on the adopted doping scheme, when the modulator is working, a positive voltage needs to be applied to the signal electrode to make the PN junction work under reverse bias conditions to achieve high-speed response. Near the output of the modulator, TiN heaters are adopted on the waveguides to precisely control the operating point of the modulator to be centered at the quadrature point.

2 Design of the slow-light structure

In the modulation arms, a coupled-resonator optical waveguide (CROW) is utilized[3] to generate the slow-light effect, in which a complete resonator is constructed by a $\lambda/4$ phase shifter region with broader width and several Bragg gratings on both sides with a period of around 300 nm. The structure of slow-light waveguides is demonstrated in Fig. S2a. Here, the phase shifter is in the middle position of each resonator, and a certain equal number of period (N_p) of gratings on each side (narrower Bragg gratings) construct one side beam of a resonator, then two beams are

connected with each other by a $\lambda/4$ phase shifter region (broader part) to form a complete CROW resonator, along the direction of light propagation in the waveguide. Thus, through the $\lambda/4$ phase shifter region, the supercell can generate a topological mid-gap mode embedded in the photonic bandgap, between the antisymmetric and symmetric transverse electric bands, as the mode adopted for multi-wavelength communications in C-band, shown in Fig. S2b. Furthermore, a finite number of resonators (N_r) are cascaded together to construct a modulation arm.

As for the physical mechanism of modulation, the modulation process is still based on the plasma dispersion effect of the depletion-mode PN junction, which is suitable for industrial applications. Also, the PN junction is based on the CROW design, illustrating a periodic structure. The part above the axis of the slow-light waveguide in Fig. S2a is P-doped (5.0×10^{17}/cm^3), and the bottom part is N-doped (5.0×10^{17}/cm^3). Here, the relatively low doping concentration of PN junction is selected, which is conducive to the realization of sufficiently high electrical bandwidth due to the decrease of junction capacitance. Meanwhile, the efficiency factor (define as $\phi/\pi V$ to characterize the phase change per voltage) can be effectively compensated and controlled by introducing the slow-light waveguide structure. The zoom-in SEM image of the fabricated slow-light waveguide is also demonstrated in Fig. S2a (part of one resonator is shown here, including a $\lambda/4$ phase shifter region and six periods of Bragg gratings on both sides), with actual measured parameters (the core and overall width of the waveguide, the period and width of the gratings, the width of the phase shifter). As a one-dimensional waveguide grating structure, silicon CROW possesses high CMOS compatibility, and all feature sizes are suitable for commercial silicon photonic foundry.

The slow-light scheme provides a high degree of design freedom, and the structure parameters can be flexibly selected according to the specific application scenarios. In the slow-light design, the selection of group index is critical to the overall performance of the device. For the ultrahigh-speed applications here, if the slow-light effect is set to be over high by adjusting the grating structure, the insufficient optical bandwidth will reduce the EO bandwidth significantly, which will become the primary bottleneck. Simultaneously, the group index cannot be too low because of the limitation in modulation efficiency. Therefore, based on our optimized waveguide grating structure, the designed group index of $n_g = 6.1$ is a carefully optimal value to balance bandwidth and modulation efficiency, which is the key point for ultrahigh-speed applications in our work. Considering the group index of the typical silicon waveguide is around 4.0, the slow-light factor in the designed slow-light waveguide is 1.525. In fact, the values of N_r and N_p will directly affect the optical performance of the device, thus we can build a reconfigurable slow-light model to describe the relationships. Fig. S2c-e demonstrates the variation of optical bandwidth, efficiency factor and phase accumulation with N_r and N_p. In general, the increase of N_r and N_p, which means the enhancement of slow-light effect, will tune up efficiency factor and Q factor but reduce optical bandwidth. Therefore, it is essential to select favorable N_p and N_r to balance the parameters according to the application scenarios. For a certain N_p value of 20, Fig. S2c shows the change of optical bandwidth and efficiency factor with N_r. When N_r increases, the optical bandwidth will decrease gradually and the efficiency factor basically linearly. In contrast, for a certain N_r value of 20, the increasing N_p will enhance the efficiency factor quite obviously, especially for the region over 30 (Fig. S2d). However, higher N_p will constrain the optical bandwidth, and this

will restrict the modulator performance at ultrahigh-speed applications.

Here, to support high baud rate transmission, we focus on maintaining high bandwidth and increase the efficiency factor on this basis, so we set N_p to 20 and tune the value of N_r. For the phase accumulation, Fig. S2e illustrates the influence of increasing N_r for the phase change under different voltages (N_p maintains at 20). It can be seen that the doubled N_r (N_r 20 compared to N_r 10) will enhance the phase change accumulation greatly, which will be more effective at higher voltage. Therefore, for the goal of achieving ultrahigh-speed advanced format transmission, which requires high baud rate and sufficient modulation depth, we focus the design of $N_r = 20$ and $N_p = 20$ here, to achieve a balance between bandwidth and efficiency, thus realizing sufficient phase change accumulation while minimizing signal transmission loss. In theory, the optical bandwidth and efficiency factor for the actual designed device are 0.028 π/V and 95 GHz, respectively.

3 DNN transmission results

In the DNN, a specially designed multi-level real-value $sigmoid$ activation function is employed. The activation functions traditionally employed in artificial neural networks for signal equalization typically exhibit two saturation regions (-1, +1 or 0, 1), such as $sigmoid$, $tanh$, and $softmax$, as shown in Fig. S3a. However, in scenarios involving high-order modulation, such as PAM-4/PAM-8 in IM/DD systems, where the modulation level exceeds two, the effectiveness of ANN-based equalizers tends to be inadequate. The signal equalization process can be conceptualized as a classification problem, wherein signals are categorized into different classes, such as (-3, -1, 1, 3)

in PAM-4 or (-7, -5, -3, 1, 3, 5, 7) in PAM-8, respectively. It is crucial that the output values of the neurons in the ANN equalizer lie within the saturation region. For a high-order modulation signal, it should be classified into multiple categories at the output of the ANN equalizer. It is feasible to find an activation function featuring multiple saturation regions within a multi-level neuron. Here an activation function with four and eight saturation level regions are implemented through function $f(x)$=2η_2/(1+$e^{-\eta_1(x-2\alpha)}$)-η_2+2α, where α equals to -1, 0, 1 when x≤-1, -1<x≤1 and x>1, respectively, and η_2= (1+$e^{-\eta_1}$)/(1-$e^{-\eta_1}$). η_1 represents the gradient factor, continuity of the function is kept by η_2. The constructed $f(x)$ has four saturation regions which are close to the amplitude of PAM-4 (-3, -1, 1, 3), as shown in Fig. S3b. Similarly, it can be extended to 8-level sigmoid function as shown in Fig. S3c which can be used to equalize the modulated signals such as PAM-8, 8-ASK. The multi-level characteristic of the activation function $f(x)$ makes it suitable for PAM-4 and PAM-8 equalization, which has not been explored in the form of deep neural network with multiple hidden layers in the scenario of IM/DD systems.

As a comparison, the system performance employing $tanh$ and two-level $sigmoid$ as the activation function are also measured, as shown in Fig. S4. Two groups of data (group 1 in Fig. S4a and group 2 in Fig. S4b) with different date rates (130 Gbps and 170 Gbps) of PAM-4 are selected for the measurements, the results indicate that the performance is relatively poor when using either $tanh$ or two level $sigmoid$ as activation functions. However, when a four-level $sigmoid$ function is employed, it outperforms both the $tanh$ and two-level $sigmoid$ functions. Both the $tanh$ and $sigmoid$ functions are commonly used to introduce non-linearity in deep neural networks, $tanh$ is generally favored in hidden layers for its better gradient properties, it typically has a stronger

gradient compared to the $sigmoid$ function, making it more suitable for training deep networks with many hidden layers.

Since 224 Gbps PAM-4 per lane is the IEEE standard for 1.6 TbE, we adopt the relatively simple DNN equalizer on the Si-SLM chip first, taking less computing sources. The transmission results including the eye diagrams and BERs of 224 Gbps and 200 Gbps at all 8 channels from 1548 nm to 1555 nm are demonstrated in Fig. S5. The eye diagrams are clear and all BERs for all channels are below 2×10^{-2}, leading to a total capacity of 1.6 Tbps. Furthermore, a 3.2 TbE interface can be realized by scaling out the channels based on 224 Gbps PAM-4 transmission per lane.

4 GRU transmission results

GRU networks are made up of GRU cells, which are units that contain a series of gates that can control the flow of information into and out of the cell. The gates can learn to keep relevant information and discard irrelevant information, allowing the cell to remember important information for long periods of time. However, the GRU is less complex than the LSTM, as it has only two types of gates: the reset and update gates. The reset gate is used to handle short-term memory, whereas the update gate is responsible for long-term memory. Models with bidirectional structure are capable of learning information from both preceding and following data when processing the current data. The bi-GRU model comprises two unidirectional GRU layers operating in opposite directions. One GRU processes the input sequence in the forward direction, starting from the beginning, while the

other operates in reverse, processing the sequence from the end toward the start. By combining forward and backward GRU processing, the model incorporates information from both the future and the past to influence its current states. The bi-GRU model relies on the states of two GRUs, whereas the T-biGRU model utilizes the states of three GRUs. By integrating forward, backward, and repeated forward GRU processing, the T-biGRU model more comprehensively extracts both global and local features of the sequence.

To explore the transmission potential of silicon photonics, we adopt bi-GRU equalization in neural network to push the data rate to the next stage. All the PAM-4 eye diagrams, constellations and corresponded BERs for all channels and data rates from 200 Gbps to 400 Gbps on the Si-SLM chip are summarized in Fig. S6. All the eye diagrams are quite high-quality, favorable differentiation between the four levels can be observed in the constellations and the BERs are all below HD-FEC threshold, even up to 400 Gbps. There is no obvious difference between different channels, indicating the good performance consistency. Based on 400 Gbps PAM-4 transmission per wavelength, an aggregation data rate of 3.2 Tbps is realized, with a remarkable on-chip data-rate density of 1.6 Tb/s/mm^2.

Moreover, to verify the scalability of our AI-accelerated slow-light solution for higher-order signals, the PAM-8 signal experiment is implemented based on the WDM Si-SLM chip. The data rates are set to from 240 Gbps to 390 Gbps. All the eye diagrams, constellations for all channels at different rates are demonstrated in Fig. S7, with all BERs below HD-FEC threshold. The experimental results illustrate that even for PAM-8 signals with more levels, our approach still

possesses a powerful ability for mitigating the nonlinear distortions in muti-level signals.

Furthermore, the T-biGRU transmission results are evaluated for both PAM-4 and PAM-8 signals. The T-biGRU PAM-4 results of eye diagrams, constellations and BERs for Ch2, Ch4, Ch6 and Ch8 are demonstrated in Fig. S8, from 280 Gbps to 400 Gbps. Meanwhile, the T-biGRU PAM-8 results from 300 Gbps to 390 Gbps are demonstrated in Fig. S9. The experimental results illustrate that the quality of eye diagrams and constellations is further enhanced, and the BERs for all channels can be improved to smaller than 10^{-3}, even up to around 400 Gbps, while the high consistency is maintained between different channels.

5 AI equalizer network configurations

The AI equalizer is used at the back end of the system, and the distortions suffered by signals at different baud rates can serve as an indicator of the different nonlinear distortions encountered. Based on this consideration, the system performance for different baud rate signals under different AI equalizer network configurations is evaluated.

The core advantage of the GRU algorithm is its ability to effectively capture long-range dependencies in sequential data. For the GRU equalizers, models with bidirectional structure are capable of learning information from both preceding and following data when processing the current data. The first layer is the input layer, the current symbol x_i with its k preceding and k succeeding symbols together are used as the input sequence of the network. In this work, to accurately model temporal dynamics and efficiently process the sequential information, we configured

the input layer's symbol count to match the maximum data size captured in each instance. Specifically, in the experiment each capture consists of 32767 signal symbols, corresponding to a total data size of 4.2 MB. Consequently, the input layer's symbol count is set to 32767, with k equal to 16383. When k is set to smaller values, such as 10000 and 5000, the performance is also evaluated, as shown in Fig. S10. The results indicate that these cases exhibit inferior performance compared to k=16383, which may be attributed to insufficient sequential information in the data stream. The value of k is determined by the number of symbols the equalizer can process. To retain more sequential information, larger k values are preferred.

The number of neurons and the number of layers are important factors affecting the performance of neural networks. The BER performance of the DNN equalizer initially improves for all data rates as the number of input neurons increases (Fig. 3g). However, after reaching a certain threshold, specially 25 neurons for our network, further increases do not yield additional performance gains. Meanwhile, as the number of neurons in the hidden layer increases, the BER performance does not exhibit significant (exponential-order) promotion (Fig. 3h). Furthermore, Fig. S11a illustrates the performance variation with different numbers of hidden layers. The results indicate that when the number of layers exceeds four, performance saturation occurs. Since a larger network scale does not improve system performance significantly and consumes more computational power, two-hidden-layer structure is employed in the experiment. For the GRU equalizer, with more GRU units employed in each direction of the equalizer (Fig. 4b), the BER will be better, as shown in Fig. S11b. For both the cases of 300 Gbps and 400 Gbps signal, increasing the number of GRU units could effectively reduce BER. This trend remains evident up to 230 units,

beyond which performance saturation occurs, with no significant further improvement. Another dimension is to increase the number of GRU directions in the equalizer. By further extending the T-biGRU to a dual-biGRU, which incorporates two forward and two backward directions, further performance improvements can be achieved as given in Fig. S11c.

For the training of different data groups in the experiment, different groups (Data Group 1-5) of data with the same size (more than 30k symbols each, with 4.2 MB data size) were captured from the oscilloscope and used separately for training and testing the AI equalizer. The system performance at different training data percentages in one data group, specially 30%, 50%, 80%, and 100%, is evaluated, with the results shown in Fig. S12a, where the results are the average BERs of employing other four data group as test data (DNN equalizer). The results indicate that with 80% and 100% percentage of the training data, the system performance is optima. For the main part, the results presented in Fig. 3 to Fig. 7 were obtained using 80% of the training data from a single data group, while BER represents the average results from the remaining data groups. Also, by selecting training data from one data group (Data Group 1) and testing on the remaining data groups (Data Group 2-5), the performance of different data groups is given in Fig. S12b (bi-GRU equalizer). The BER results indicate favorable consistency among different data groups.

GRUs utilize gating mechanisms to selectively retain or discard information, allowing them to effectively remember signal context, which is an essential capability for mitigating inter-symbol interference (ISI) in high-speed PAM-4 and PAM-8 transmissions. This architecture provides a parameter-efficient means of capturing temporal dependencies. Compared to feedforward DNNs,

bidirectional or multidirectional GRU models can leverage both past and future context, thereby enhancing symbol prediction accuracy. Here, the general design principles for developing a GRU-based optical equalizer are summarized. For the input window, which provides temporal information the GRU needs to model ISI, the input typically includes k preceding and k succeeding symbols around the current sample. The value of k is a hyperparameter and relates to channel memory length, and larger k values are preferred to retain more sequential information. In the network architecture, one or more layers of GRU units can be configured, and multi-directional processing can be employed to capture both past and future contextual information within the sequence. Employing a relatively lite architecture is recommended to achieve an optimal balance between performance and power consumption. Also, it is important to select an appropriate loss function based on the output structure. Typically, mean squared error (MSE) or cross-entropy loss can be used depending on the properties of the network output. The network is trained to minimize the discrepancy between the predicted symbols and the actual transmitted symbols by optimizing a predefined loss function. For the training strategy, a large labeled dataset of received symbols and corresponding transmitted symbols can be used to train the equalizer. By introducing variations in the training dataset, the generalization of the equalizer can be enhanced. Meanwhile, determining equalization performance evaluation metrics such as BER is necessary for assessing the effectiveness of the equalizer in mitigating signal impairments, and ensure that the model is deployable under given hardware constraints while recording the computational and memory overhead during both training and inference stages.

6 Performance comparison between equalizers

In most transmission scenarios, signal equalization is essential for high-order modulation formats such as PAM-4 and PAM-8. The linear equalizers such as feed-forward equalization (FFE) and decision-feedback equalization (DFE) are effective methods for linear impairments compensation and widely used nowadays[4]. The most basic component of FFE is the finite impulse response (FIR) filter, the output of FIR is expressed as:

$$z(k) = \sum_{l=0}^{n-1} h_l x(k-l) \tag{S1}$$

where $x(k)$ and $z(k)$ are the input and output signal of FIR at the sampling instant k, respectively. $h = [h_0, h_1, h_2 ... h_{n-1}]$ is the array of tap weights, while n is the number of taps. Unlike FFE, the input of DFE is the signal after decision, the output signal of DFE can be calculated as:

$$z(k) = r(k) - \sum_{l=0}^{n-1} h_l \delta(k-l) \tag{S2}$$

By adopting FFE/DFE, the linear impairments can be eliminated efficiently. However, the residual nonlinear distortions mainly induced by the device and system can severely impact the transmission performance, which cannot be effectively compensated by FFE/DFE. One of the most common algorithms to compensate nonlinear effects is Volterra nonlinear equalizer (VNLE)[5], the output of third-order VNLE can be expressed as:

$$z(k) = \sum_{l=0}^{n_1-1} h_l x(k-l) + \sum_{l=0}^{n_2-1} \sum_{i=0}^{l} h_{l,i} x(k-l)x(k-i) + \sum_{l=0}^{n_3-1} \sum_{i=0}^{l} \sum_{j=0}^{i} h_{l,i,j} x(k-l)x(k-i)x(k-j) \tag{S3}$$

where h_l, $h_{l,i}$, $h_{l,i,j}$ are the tap weights of 1st-order, 2nd-order, 3rd-order kernels, respectively. n_1, n_2 and n_3 respectively represent the number of taps for the linear part, 2nd-order nonlinear part and

3rd-order nonlinear part. Linear impairments in the system can be eliminated by the first-order kernel of VNLE, while the second-order and third-order kernels compensate for nonlinear distortions. VNLE expands the equalization function using polynomial terms to model nonlinear distortions, the computational complexity grows exponentially in VNLE with the order of nonlinearity increases. The neural network is computational model loosely inspired by its biological counterparts and has been proposed to mitigate the nonlinear impairment in optical system. By training on large datasets, neural network can better capture the underlying distribution of the optical signal and learn the subtle relationships between different symbols, this enables them to effectively handle cases where traditional equalizers might fail. Neural networks can learn to directly map the distorted signal back to the transmitted symbols without manually decomposing the problem into individual components, which shows a completely different work philosophy compared to DFE and VNLE that based on filters.

To further measure the difference between AI equalizers and traditional algorithms, the performance of DFE and VNLE compared to DNN, bi-GRU and T-biGRU for high-speed PAM-4 and PAM-8 signals of different rates is evaluated, the results are shown in Fig. S13. The tap weights of DFE are updated by least mean squares (LMS) algorithm and third order VNLE is employed. The results indicate that by employing bi-GRU and T-biGRU, compared to DFE and VNLE, there is obvious performance promotion at 300 Gbps, 360 Gbps, 400 Gbps PAM-4 (Fig. S13a) and 330 Gbps, 360 Gbps, 390 Gbps PAM-8 (Fig. S13b) signals, respectively. From the experimental results, it can be seen that AI equalizers outperform both DFE and VNLE.

7 Robustness of the neural network

In real transmission scenarios, if the transmitter output intensity varies due to changes in link conditions (e.g., power fluctuations, modulation depth variations, or environmental factors), a neural network equalizer may require retraining or adaptation. Variations in output intensity can affect signal-to-noise ratios (SNRs), modulation depth, and potentially signal shapes. This can change the distribution of the transmitted symbols, on which the neural network was initially trained. If the model encounters a signal that deviates from the training distribution (e.g., due to lower intensity or clipping), its performance could degrade, as it may not effectively map the received signal to the correct symbols.

Improving the robustness of the AI equalizers involves several key strategies: 1) Data mixing or augmentation. By introducing variations in the training dataset to enhance generalization, such as incorporating data from different power levels, SNRs and nonlinear distortions; 2) Apply dropout or $L2$ regularization to prevent the neural network overestimating and encourage robustness to variations in input signals; 3) Combine neural networks with traditional signal equalization methods to enhance interpretability and robustness. Additionally, training the network with adversarial examples or utilizing transfer learning by pretraining the model on a broader dataset can further enhance its resilience to unexpected distortions. However, this process may require a significant amount of time to obtain sufficient and useful training data.

We train the DNN network with mixed data that captured at different power -15.87 dBm, -16.75 dBm, -17.78 dBm, -18.70 dBm and -19.77 dBm with the same proportion (2:2:2:2:2), and

then test the signal performance of each power, the results for 130 Gbps PAM-4 under DNN are shown in Fig. S14. The results indicate that training the network using mixed data from different powers and then testing at other power yields better performance than training at a specific fixed power and then testing at other power. However, compared to retraining the network separately at each power level and then testing at each corresponding power, the performance remains inferior.

We also measure the bi-GRU performance for 280 Gbps PAM-4 training at a specific fixed power and then testing at other power, the results are given in Fig. S15a. The results show that there is obvious performance degradation when employing the network trained at a specific fixed power to test the data from other power compared to retraining the equalizer at each power separately. However, it is still better than the DNN case with fixed trained power, which shows the better robustness of bi-GRU to power variations. The results of training the bi-GRU using mixed data captured at different power levels and then testing on other power levels are also shown in Fig. S15b. By training the model with mixed data from different powers, the overall BER performance can be improved to some extent compared to the training based on a specific fixed power.

On the other hand, dropout randomly deactivates a fraction of neurons during training, forcing the network to rely on different subsets of features in each training iteration, during inference (when dropout is turned off), the full network acts as an ensemble of many smaller networks, leading to improved performance and robustness[6–8]. For tasks like optical signal equalization, where the input data can be affected by noise, dispersion, and nonlinear distortions, dropout helps the network learn features that are less sensitive to these variations. This prevents the model from

becoming too dependent on specific neurons and memorizing the training data, improving generalization to new data. By adding dropout layer, we further give a test to the performance of employing dropout to bi-GRU for 280 Gbps PAM-4, the results are shown in Fig. S16a. The results indicate that incorporating dropout in the network leads to better performance compared to the case without dropout.

In the meanwhile, $L2$ regularization (also known as weight decay) is a technique used to prevent overestimating by penalizing large weights in a neural network[9,10]. It modifies the loss function by adding a penalty term proportional to the sum of the squared weights, encouraging the model to keep weights small and thus improving generalization. We also tried employing $L2$ regularization to the bi-GRU network for 280 Gbps PAM-4, the results are shown in Fig. S16b, and the improved performance proves the effectiveness of the method.

Furthermore, by applying both dropout and $L2$ regularization to the bi-GRU network simultaneously, the performance demonstrates superior results compared to using either method individually, indicating further enhanced network robustness to power variations, as shown in Fig. S16c. However, the performance remains inferior compared to retraining the model at each specific power level separately.

By employing a relatively lite AI equalization method within the proposed system, a balance between performance and power consumption can be achieved. Therefore, the employed network scale of the AI equalizer in this work is small, only consisting of four layers in total for the DNN and three layers for the GRU, respectively. Normally, for neural networks, smaller network scale

tends to exhibit lower robustness, which has been shown by numerous studies. Under the network configuration shown in this work, the robustness is still challenging. By employing data mixing, dropout and $L2$ regularization, the robustness can be enhanced to some extent.

8 Power consumption

To evaluate the power consumption of Si-SLMs (excluding systems), we have calculated the energy consumption per bit (E_b) of Si-SLMs first, which is dominated by the termination resistors[11]. For push-pull driving, the power dissipation (P_T) at the termination resistors (R_T) is given by:

$$P_T = 2 \times \frac{(\frac{V_{pp}}{2})^2}{R_T} \tag{S4}$$

Based on this, the P_T is calculated to be 0.454 W, and the corresponding E_b is 1.135 pJ/bit at 400 Gbps. Although the relatively high V_{pp} value results in a power increase, the enhanced transmission speed allows the E_b of Si-SLMs to remain at an acceptable level.

For the practical system, in this proof-of-concept demonstration, we evaluated the power consumption of each key component involved in the experiment. Although not optimized for energy efficiency, this provides a reference scenario that can be used as a benchmark for future integration and optimization designs. In the experiment, the bench-top laser source provides the optical carrier signal with the output power of 13 dBm. The maximum apparent power consumption of this laser is 100 VA, with real power consumption not exceeding 100 W. For the modulation part, the main power consumption includes the DC bias, TiN heater, and drivers. The recorded voltage and current values in the experiment process allowed us to calculate the actual system power consumption.

By reading the actual voltage and current values directly from all equipment ports, the calculated power consumption includes contributions from the PN junctions, termination resistors, and TiN heaters of the modulator. For DC bias, two modulation arms were given a DC bias of 2.5 V each with the measured currents of 0.077 A and 0.080 A, resulting in a power consumption of 0.393 W. To ensure that the modulator operates at the quadrature point, one TiN heater was driven at 2.290 V and 0.011 A, thereby the corresponding power consumption was 0.025 W. For the electrical amplifiers, two drivers were connected in parallel to the same power supply channel. The measured operating voltage and current were 9 V and 0.531 A, thus the combined power consumption of drivers was 4.779 W. It should be noted that we did not employ additional thermoelectric cooling (TEC) for the modulator due to the intrinsic thermal stability of the Si-SLM. For the optical amplifier, a discrete erbium-doped fiber amplifier (EDFA) was placed before the photodetector (PD) to enhance the input optical power. The conventional discrete EDFA with similar gain and noise has a maximum power consumption of about 20 VA, with real power not exceeding 20 W. For simplicity and comparative purposes, we set the EDFA power consumption to approximately 20 W. For the AI equalizers, referring to the prior measurement work[12], the power consumption was monitored using HWINFO, ranging from 35 W for the DNN to 67 W for bi-GRU and T-biGRU implementations. Summing these contributions, the total system power consumption varied from approximately 159.887 W to 192.757 W, depending on the chosen AI equalizer. And all these values are listed in Table S1.

Furthermore, we evaluated and compared the power consumption of traditional nonlinear algorithm and AI equalizers. By employing VNLE, we achieve a compatible BER below HD-FEC

threshold at 130 Gbps PAM-4. Using the same method as described above for AI equalizers, the power consumption was measured to be 42.39 W during the VNLE algorithm running. Here we define the equalizer power consumption per bit ($EPCpB$) as:

$$EPCpB = EqualizerPowerConsumption(W)/DataRate(Gbps) \tag{S5}$$

A smaller $EPCpB$ value indicates a lower power consumption per bit for equalizers. Based on the power consumption of each equalizer, the calculated $EPCpB$ for VNLE, DNN, bi-GRU, T-biGRU are shown in Table S2, where the speed selections are all below HD-FEC threshold. From the calculation results, it can be seen that the speed reduction of employing VNLE does not justify the decrease in energy usage. Simultaneously, under the circumstance that the algorithm is already adopted, the AI equalizers (especially bi-GRU and T-biGRU) have not significantly increased $EPCpB$ (and in some cases, even reduce it). This is because the increase in power consumption also brings about an enhancement in the transmission rate, so that the energy consumption of algorithm required to transmit each bit of data is controlled within an acceptable range. In other words, it is worthwhile to use higher equalizer power consumption in exchange for an improvement in transmission speed based on AI equalizers. This result proves the feasibility of the proposed solutions in terms of equalizer power consumption.

It is important to reiterate that our experiments are still at an early proof-of-concept stage, aimed at demonstrating the potential of Si-SLMs for ultra-high-speed, large-throughput signal transmission. It is anticipated that ongoing advancements, such as on-chip frequency combs[13–15], integrated optical amplifiers[16,17], and monolithic integration of light sources[18], modulators, and drivers[19], will substantially reduce these indicators. Likewise, techniques like network pruning[20],

quantization[21], and distillation[22], as well as hardware-accelerated inference[23], are expected to lower AI equalization power. Overall, as device integration and algorithmic optimization advance, the energy efficiency of Si-SLMs for ultrahigh-speed data center interconnections can be improved dramatically.

1. Kharel, P., Reimer, C., Luke, K., He, L. & Zhang, M. Breaking voltage–bandwidth limits in integrated lithium niobate modulators using micro-structured electrodes. *Optica* **8**, 357–363 (2021).

2. Ding, R. *et al.* High-speed silicon modulator with slow-wave electrodes and fully independent differential drive. *Journal of Lightwave Technology* **32**, 2240–2247 (2014).

3. Yariv, A., Xu, Y., Lee, R. K. & Scherer, A. Coupled-resonator optical waveguide: a proposal and analysis. *Optics Letters* **24**, 711–713 (1999).

4. Zhong, K., Zhou, X., Huo, J. & et al. Digital signal processing for short-reach optical communications: A review of current technologies and future trends. *Journal of Lightwave Technology* **36**, 377–400 (2018).

5. Mathews, V. J. Adaptive polynomial filters. *IEEE signal processing magazine* **8**, 10–26 (1991).

6. Liu, C. *et al.* 81-GHz W-band 60-Gbps 64-QAM wireless transmission based on a dual-GRU equalizer. *Optics Express* **30**, 2364–2377 (2022).

7. Hu, X., Huo, Y., Dong, X., Wu, F. Y. & Huang, A. Channel prediction using adaptive bidirectional GRU for underwater MIMO communications. *IEEE Internet of Things Journal* **11**, 3250–3263 (2023).

8. Freire, P., Manuylovich, E., Prilepsky, J. E. & Turitsyn, S. K. Artificial neural networks for photonic applications—from algorithms to implementation: tutorial. *Advances in Optics and Photonics* **15**, 739–834 (2023).

9. Srivallapanondh, S. *et al.* Parallelization of recurrent neural network-based equalizer for coherent optical systems via knowledge distillation. *Journal of Lightwave Technology* **42**, 2275–2284 (2024).

10. Xu, Z., Ji, T., Wu, Q. & et al. Advanced Neural Network-Based Equalization in Intensity-Modulated Direct-Detection Optical Systems: Current Status and Future Trends. *Photonics* **11**, 702 (2024).

11. Kawahara, K. *et al.* High-speed, low-voltage, low-bit-energy silicon photonic crystal slow-light modulator with impedance-engineered distributed electrodes. *Optica* **11**, 1212–1219 (2024).

12. Bai, B. *et al.* Microcomb-based integrated photonic processing unit. *Nature Communications* **14**, 1–10 (2023).

13. Shu, H. *et al.* Microcomb-driven silicon photonic systems. *Nature* **605**, 457–463 (2022).

14. Shu, H. *et al.* Microcomb technology: from principles to applications. *Photonics Insights* **3**, R09–R09 (2024).

15. Chang, L. *et al.* Ultra-efficient frequency comb generation in AlGaAs-on-insulator microresonators. *Nature Communications* **11**, 1–8 (2020).

16. Liu, Y. *et al.* A photonic integrated circuit–based erbium-doped amplifier. *Science* **376**, 1309–1313 (2022).

17. Davenport, M. L. *et al.* Heterogeneous silicon/iii–v semiconductor optical amplifiers. *IEEE Journal of Selected Topics in Quantum Electronics* **22**, 78–88 (2016).

18. Stern, B., Ji, X., Okawachi, Y., Gaeta, A. L. & Lipson, M. Battery-operated integrated frequency comb generator. *Nature* **562**, 401–405 (2018).

19. Li, K. *et al.* An integrated CMOS–silicon photonics transmitter with a 112 gigabaud transmission and picojoule per bit energy efficiency. *Nature Electronics* **6**, 910–921 (2023).

20. Han, S., Pool, J., Tran, J. & Dally, W. Learning both weights and connections for efficient neural network. *Advances in neural information processing systems* **28** (2015).

21. Rastegari, M., Ordonez, V., Redmon, J. & Farhadi, A. Xnor-net: Imagenet classification using binary convolutional neural networks. In *European conference on computer vision*, 525–542 (Springer, 2016).

22. Furlanello, T., Lipton, Z., Tschannen, M., Itti, L. & Anandkumar, A. Born again neural networks. In *International conference on machine learning*, 1607–1616 (PMLR, 2018).

23. Chetlur, S. *et al.* cudnn: Efficient primitives for deep learning. *arXiv preprint arXiv:1410.0759* (2014).

Table S1: | **Summary of total power consumption**

**Components**	**Voltage (V)**	**Current (A)**	**Power (W)**
Laser	N/A	N/A	$\sim$100.000
DC bias	2.500	0.157	0.393
TiN heater	2.290	0.011	0.025
Driver	9.000	0.531	4.779
EDFA	N/A	N/A	$\sim$20.000
DNN	N/A	N/A	$\sim$34.690
bi-GRU	N/A	N/A	$\sim$66.570
T-biGRU	N/A	N/A	$\sim$67.560
Total Power Consumption			Min $\sim$159.887<br>Max $\sim$192.757

Table S2: | **Summary of power consumption for different equalizers**

Equalizer	Power (W)	Signal format	BER threshold	Speed (Gbps)	EPCpB (nJ/bit)
VNLE	42.39	PAM-4	HD-FEC	130	0.3261
DNN	34.69	PAM-4	HD-FEC	170	0.2041
bi-GRU	66.57	PAM-4	HD-FEC	400	0.1664
T-biGRU	67.56	PAM-4	HD-FEC	400	0.1689

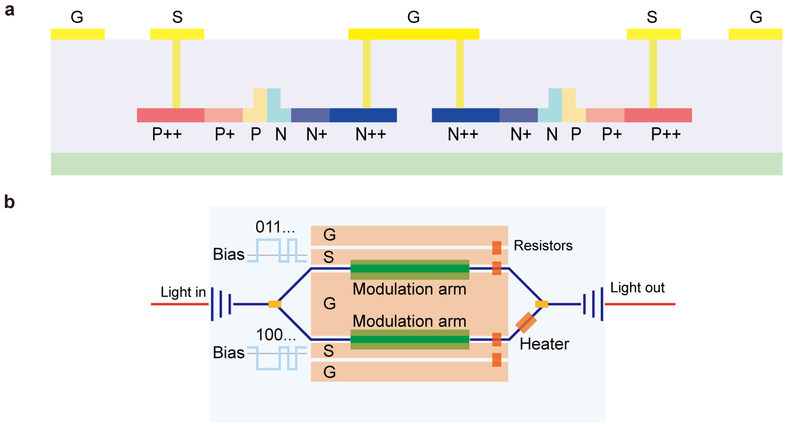

Fig. S1: | **Architecture of the silicon slow-light modulator**. (a) Cross-section view of the designed dual-drive Si-SLM. (b) Top view of the Si-SLM structure.

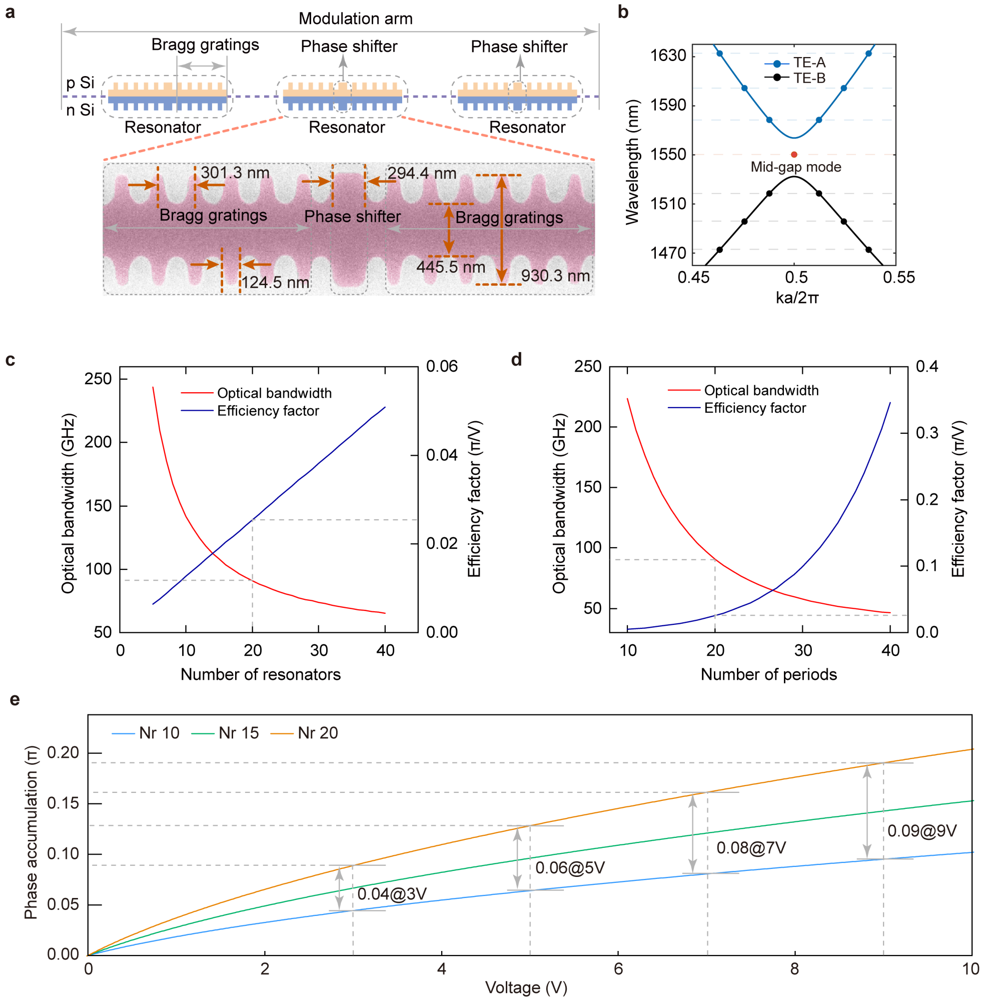

Fig. S2: | **Slow-light design model**. (a) Slow-light waveguide structure in the modulation arm. The SEM image is illustrated with actual fabricated parameters. (b) Photonic bandgap of the designed slow-light structure. The mid-gap mode is generated around 1550 nm. (c) The change of optical bandwidth and efficiency factor with number of resonators. (d) The change of optical bandwidth and efficiency factor with number of periods. (e) The influence of increasing resonator number for the phase accumulation under different voltages.

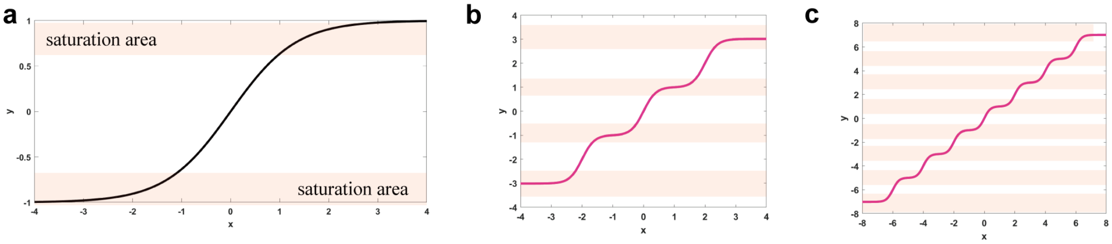

Fig. S3: | **Sigmoid functions with** (a) two-level, (b) four-level, (c) eight-level saturation regions.

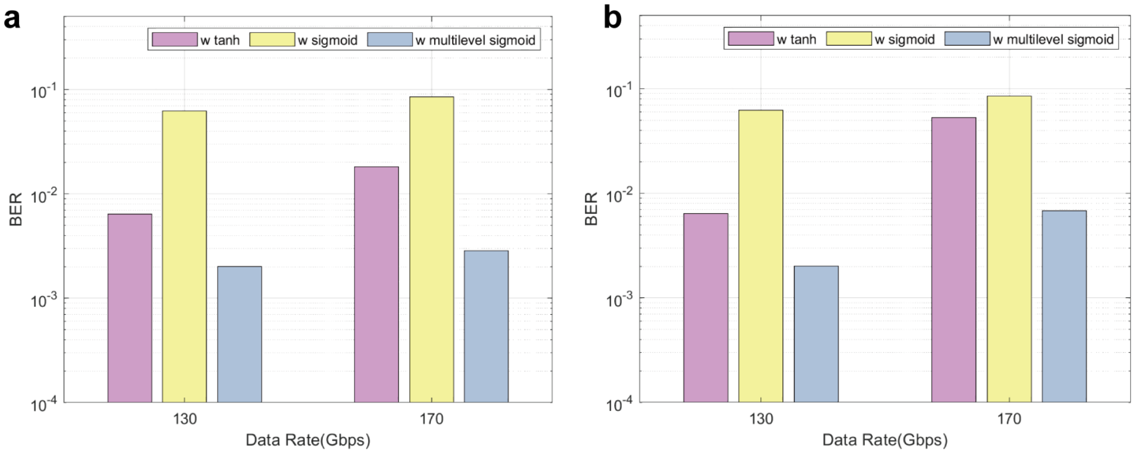

Fig. S4: | **System performance of employing tanh, two-level sigmoid and multilevel sigmoid for two groups of data with different date rates**. (a) data group 1. (b) data group 2.

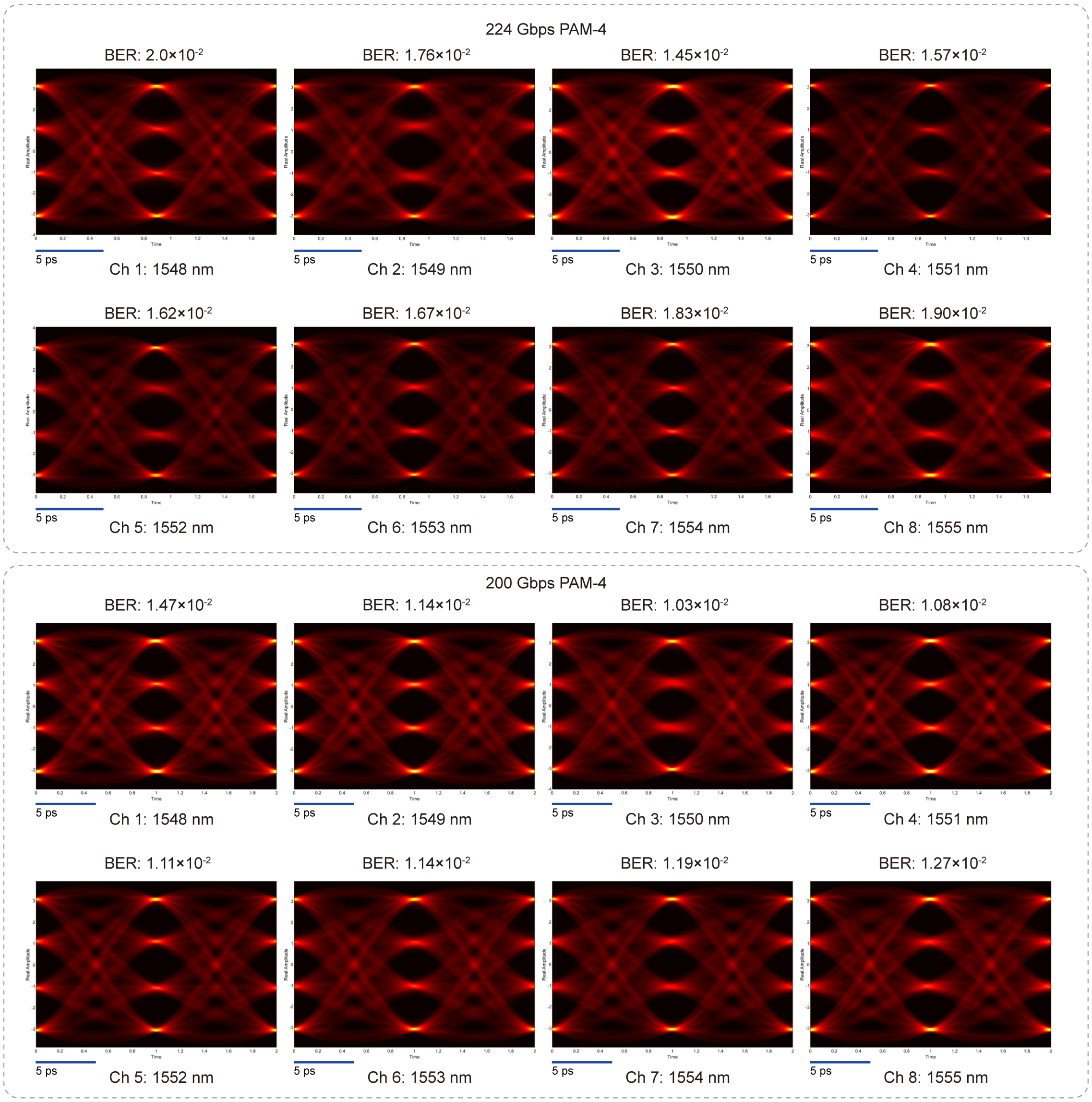

Fig. S5: | **DNN transmission results of PAM-4 signal**.

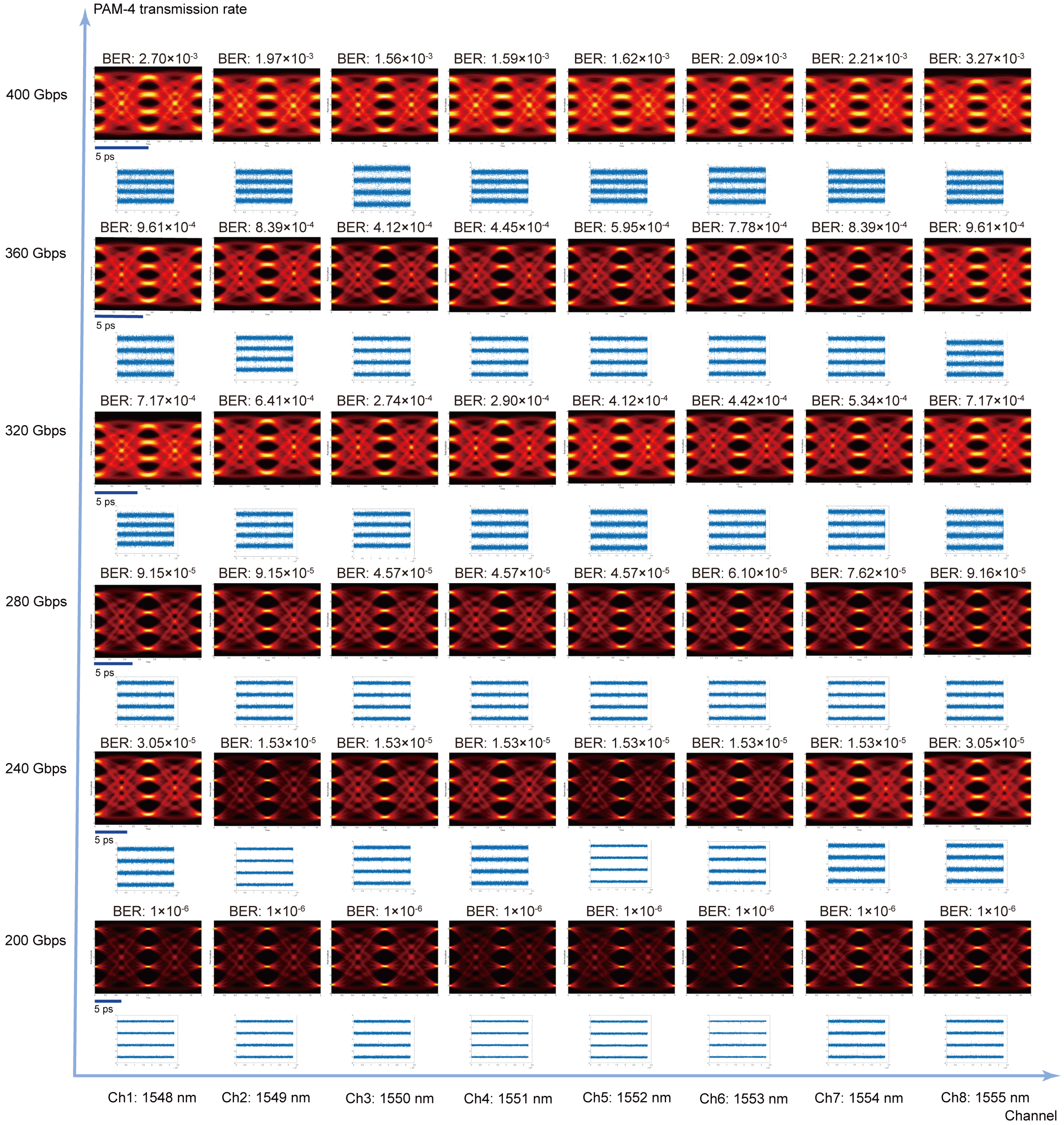

Fig. S6: | **bi-GRU transmission results of PAM-4 signal**.

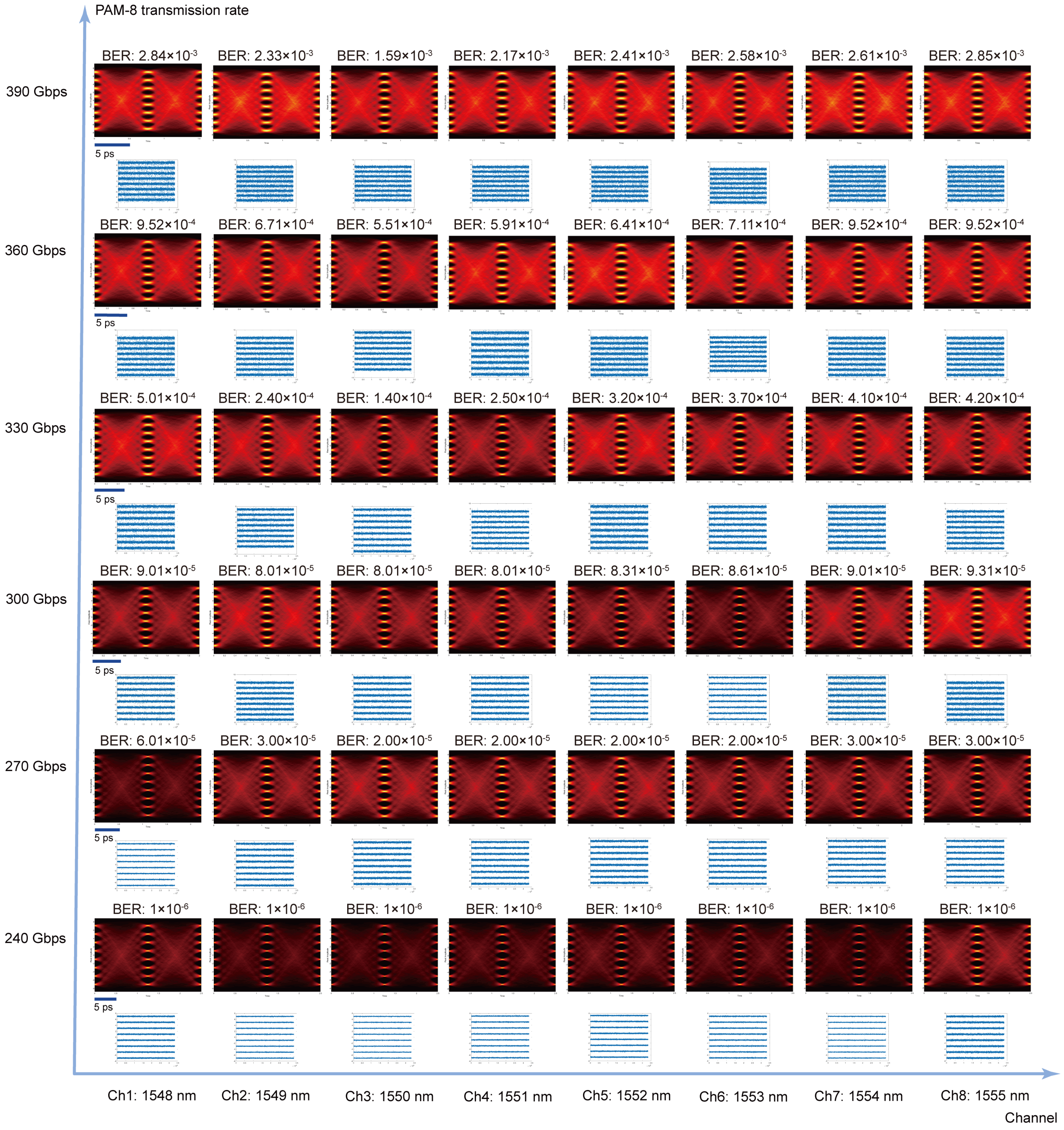

Fig. S7: | **bi-GRU transmission results of PAM-8 signal**.

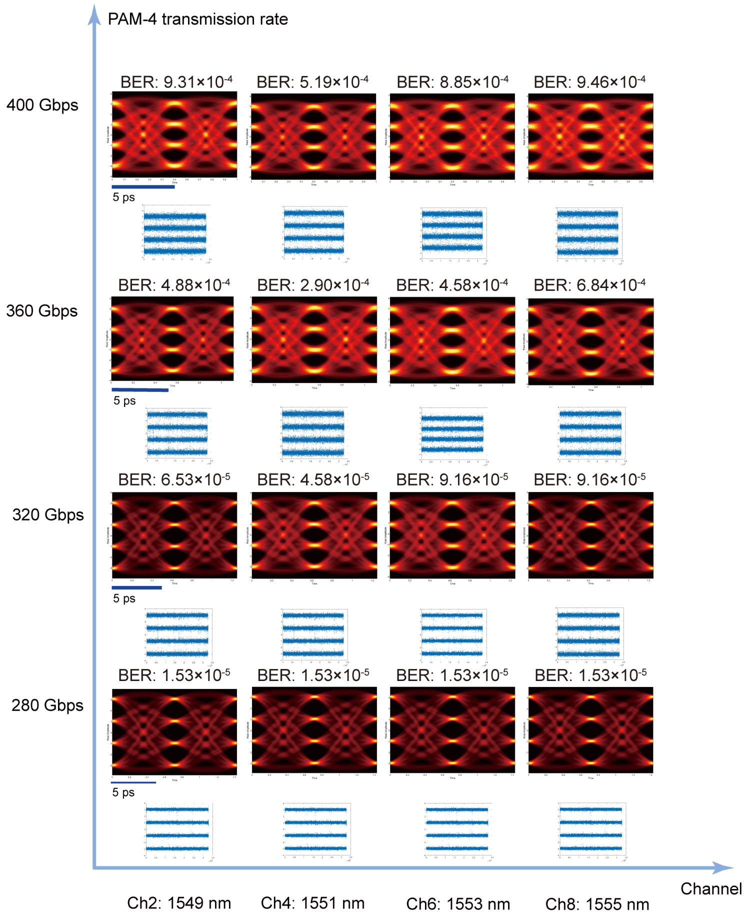

Fig. S8: | **T-biGRU transmission results of PAM-4 signal**.

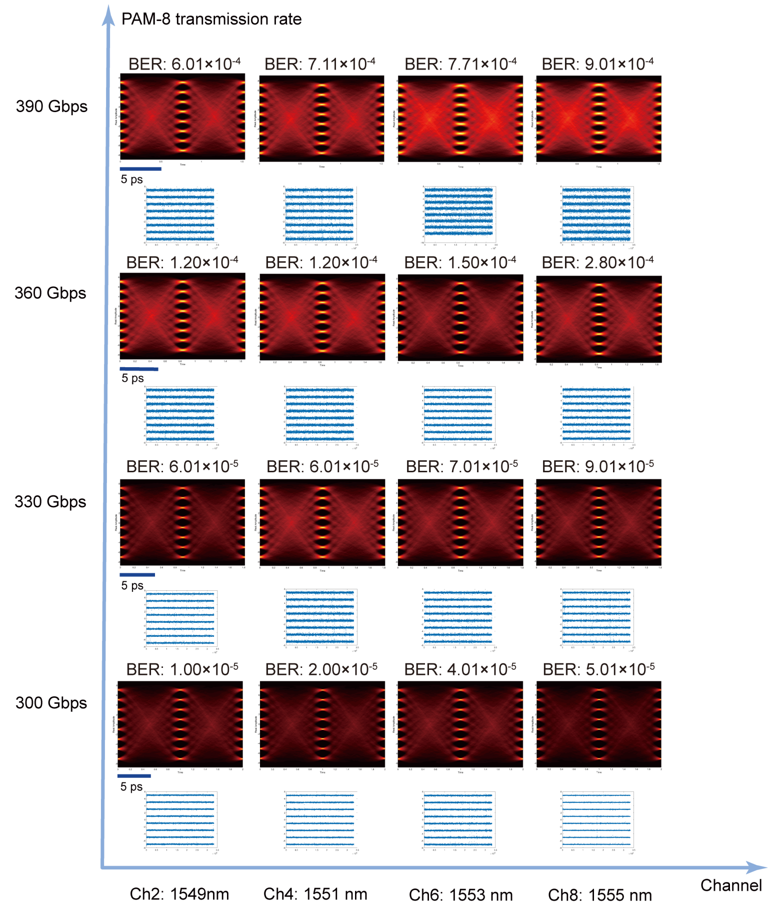

Fig. S9: | **T-biGRU transmission results of PAM-8 signal**.

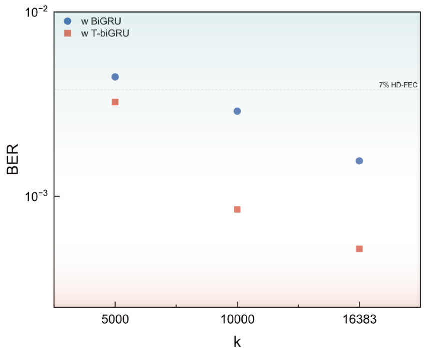

Fig. S10: | **System performance of employing bi-GRU and T-biGRU with different k values**.

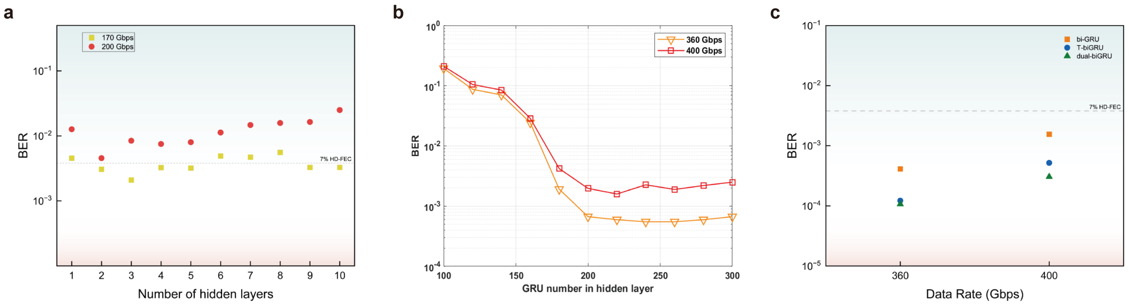

Fig. S11: | **System performance under different AI equalizer configurations**. (a) number of hidden layers in DNN. (b) number of GRU units in hidden layer. (c) number of GRU directions.

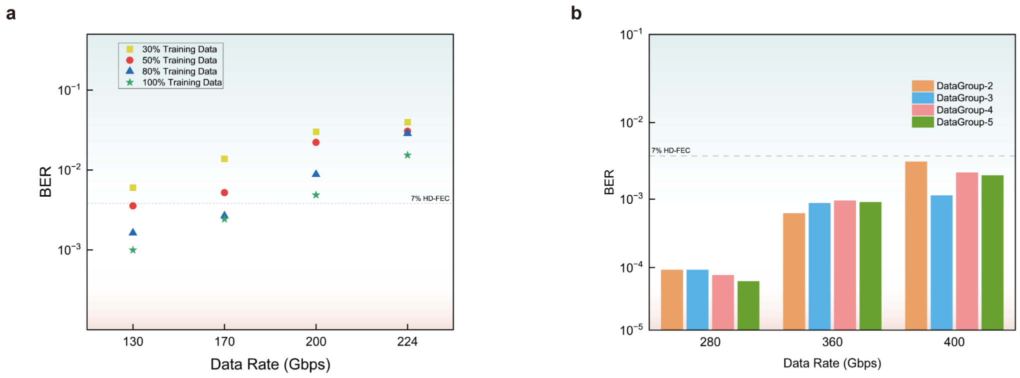

Fig. S12: | **System performance at different training data percentages and data groups**. (a) different training data percentages from one data group. (b) different data groups under the same training data.

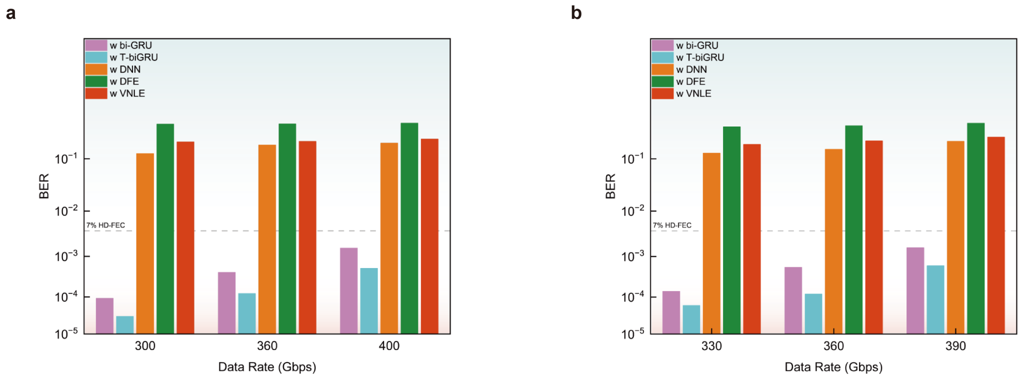

Fig. S13: | **System performance of DFE and VNLE compared to DNN, bi-GRU and T-biGRU**. (a) PAM-4 signals. (b) PAM-8 signals.

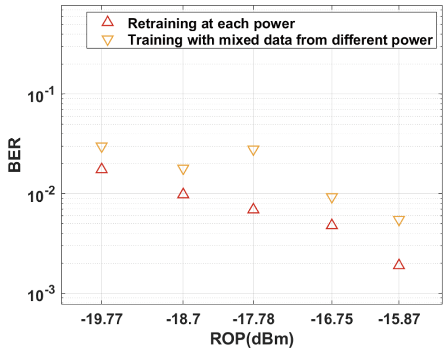

Fig. S14: | **Comparison between training DNN with mixed data from different power and retraining at each power separately**.

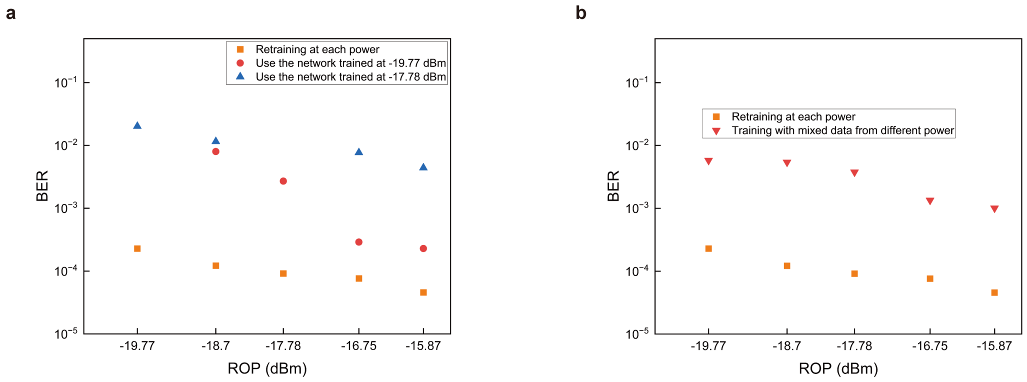

Fig. S15: | **Training bi-GRU** (a) at different fixed powers. (b) with mixed data from different power. Retraining at each power separately is also shown.

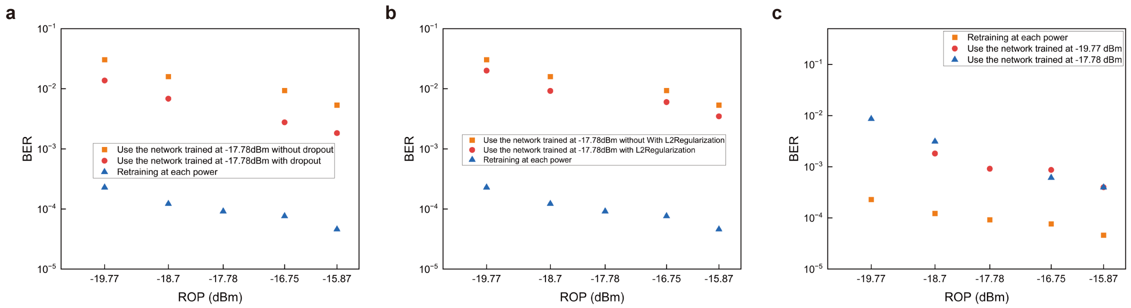

Fig. S16: | **Performance of employing** (a) dropout. (b) $L2$ regularization. (c) dropout and $L2$ regularization to bi-GRU.
